# Stakeholder engagement in developing a father-inclusive early life obesity prevention intervention: *First Heroes*

**DOI:** 10.1186/s12884-022-04759-z

**Published:** 2022-05-27

**Authors:** Santana R. Silver, Rachel C. Whooten, Gracia M. Kwete, Haley Farrar-Muir, Rachel N. Cournoyer, Elizabeth A. Barth, Milton Kotelchuck, Elsie M. Taveras

**Affiliations:** 1 Department of Social and Behavioral Sciences, Harvard T.H. Chan School of Public Health, Boston, MA USA; 2 Department of Pediatrics, Division of General Academic Pediatrics, Massachusetts General Hospital for Children, 125 Nashua St, Suite 860, Boston, MA 02114 USA; 3 Kraft Center for Community Health, Massachusetts General Hospital, Boston, MA USA; 4 Department of Nutrition, Harvard T.H. Chan School of Public Health, 665 Huntington Avenue, Boston, MA 02115 USA

**Keywords:** Fatherhood, Stakeholder engagement, Community engagement, Obesity prevention, Social determinants of health, Maternal-child health, Perinatal health, Infancy

## Abstract

**Background:**

Although paternal involvement in the perinatal period is associated with benefits for maternal-child health and reduced obesity risk, fathers are seldom included in perinatal or obesity prevention efforts. Engaging community leaders and fathers as stakeholders in intervention development is a critical step in designing a father-inclusive intervention that is efficacious and responsive to their needs.

**Methods:**

We conducted a structured engagement study, including community stakeholder engagement and qualitative interviews with new fathers, to inform the development of a prospective randomized controlled trial that includes mothers and fathers as equal partners in infant obesity prevention. We interpreted stakeholder feedback through the Consolidated Framework for Implementation Research (CFIR) framework.

**Results:**

Between September 2019 and April 2020, we held a Community Engagement meeting, formed a Community Advisory Board, and conducted 16 qualitative interviews with new fathers. Stakeholder engagement revealed insights across CFIR domains including intervention characteristics (relative advantage, complexity, design quality & packaging), outer setting factors (cosmopolitanism and culture), individual characteristics (including self-efficacy, state of change, identification with the organization) and process (engagement and adaptation). Stakeholders discussed the diverse challenges and rewards of fatherhood, as well as the intrinsic paternal motivation to be a loving, supportive father and partner. Both community leaders and fathers emphasized the importance of tailoring program delivery and content to meet specific parental needs, including a focus on the social-emotional needs of new parents.

**Conclusions:**

A structured process of multidimensional stakeholder engagement was successful in improving the design of a father-inclusive perinatal obesity prevention interventions. Father engagement was instrumental in both reinforcing community ties and increasing our understanding of fathers’ needs, resulting in improvements to program values, delivery strategies, personnel, and content. This study provides a practical approach for investigators looking to involve key stakeholders in the pre-implementation phase of intervention development.

**Trial registration:**

ClinicalTrials.gov, NCT04477577. Registered 20 July 2020.

**Supplementary Information:**

The online version contains supplementary material available at 10.1186/s12884-022-04759-z.

## Introduction

Childhood obesity is a major public health concern, with over 10% of two-to-five-year-old children in the United States meeting criteria for obesity and higher rates among children from racial/ethnic minorities and low-income families [1, 2]. Disparities in obesity prevalence originate before birth and are exacerbated by risk factors during infancy and early childhood, which influence health outcomes across the life course [3]. While early life obesity interventions are a promising strategy for obesity prevention [4], the majority target mothers and largely ignore the important role of fathers [5, 6].

Paternal engagement in early life is associated with positive maternal-infant health outcomes [7–10] and overall child well-being [11, 12]. Increasing evidence also highlights the important role of fathers in relation to childhood obesity risk [13, 14]. A father’s own obesity status and health behaviors are associated with a child’s risk of obesity, independent of maternal factors [14–16]. This may occur through several mechanisms. Fathers influence their child’s nutrition, in relation to early childhood feeding practices [17], food and beverage intake [18–20], and overall food parenting practices (i.e. access to healthy foods, modeling healthy behaviors) [9, 21]. Although less research exists specifically related to fathers and physical activity [22], there is a strong argument for a critical role for father in physical activity promotion [23, 24].

Despite this importance, barriers at multiple levels prevent adequate outreach and engagement of fathers in both early life [9, 25, 26] and obesity prevention programming [27]. These barriers include both inner setting factors, such as lack of conceptual engagement, inadequate father-focused materials and programs, and lack of trained staff to work with fathers [28], as well as outer setting factors, such as insufficient funding and lack of established best practices. On a larger scale, there is also the need for a cultural shift in recognizing the importance of fathers as partners in parenting [29]. To overcome these obstacles and meaningfully involve fathers in early life interventions, engaging key stakeholders—especially fathers—is a critical strategy to inform the design and implementation of an efficacious program that are responsive to their unique needs, perspectives, and experiences [30, 31].

The purpose of this engagement study was to engage both fathers and community stakeholders to inform the adaptation of the “*First 1,000 Days”* intervention, an evidence-based, systems-level obesity prevention program that originally targeted the mother-infant dyad, to fully involve fathers [32]. The *“First 1,000 Days”* program included universal screening of social and behavioral needs early in pregnancy and after birth, clinician/staff training on health promotion, multimedia educational materials supporting health behavior change and social needs, and individualized health coaching for women at high risk of obesity or depression. Program participation was associated with reduced risk of gestational weight gain [33], improved health behaviors and psychosocial outcomes during pregnancy [34], and improvements in both infant weight status and maternal postpartum care at 12 months of age [35].

Our goal was to engage fathers and apply their lived experiences to identify and dismantle traditional barriers preventing father engagement in the perinatal period. Through strengthening our program to meet the needs of fathers, our long-term aim is to empower fathers in promoting strategies for preventing childhood obesity. Advancing the development of informed father-inclusive perinatal programs, we hope our program can serve as a practical model for other groups that seek to incorporate both parents equally in traditionally maternally oriented spaces [36]. This manuscript describes the process and results of our stakeholder engagement.

## Methods

### Overview

**Fig. 1:**
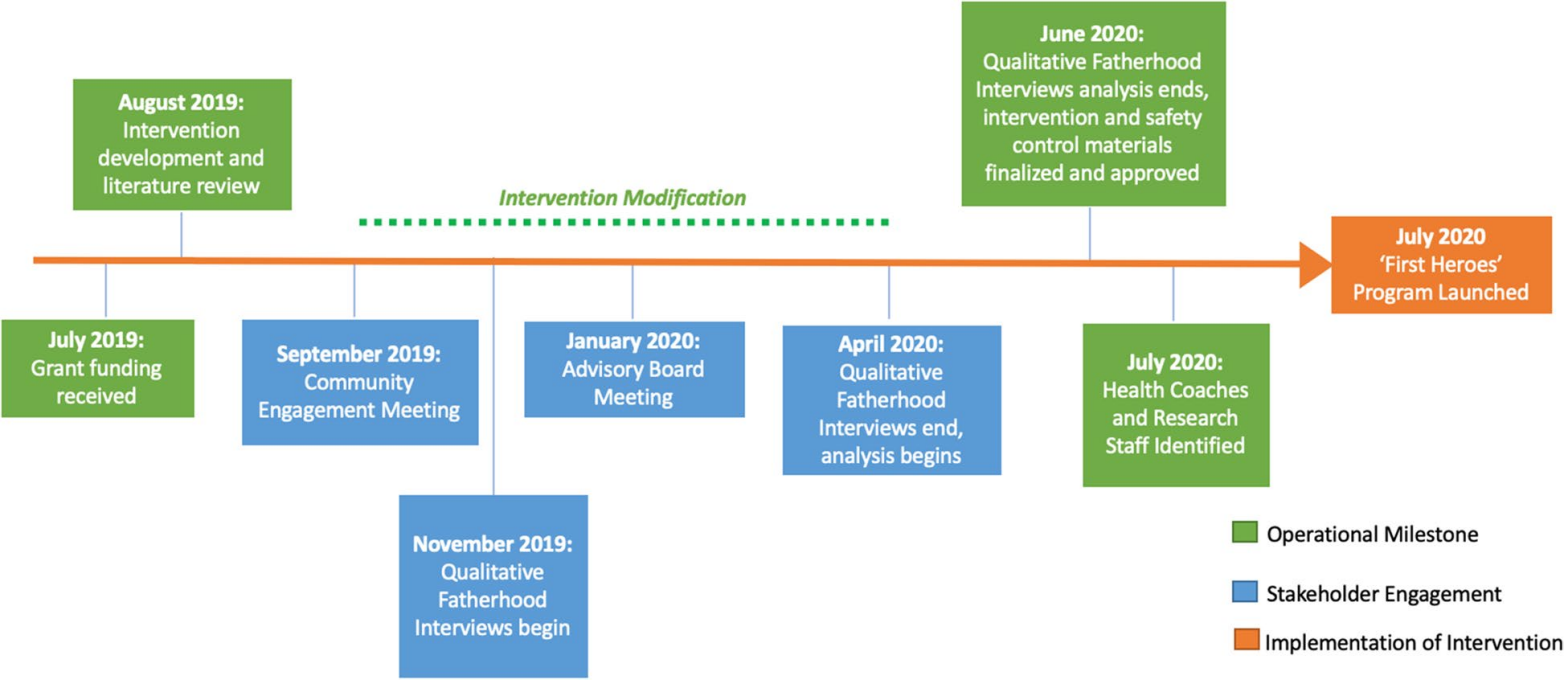
Process of Stakeholder Engagement in Design of the “First Heroes” Intervention

In planning for a new, father-inclusive intervention, we conducted a structured multilevel engagement study to identify strategies to recruit, retain, and influence fathers in perinatal and obesity prevention programs. We used the Consolidated Framework for Implementation Research (CFIR), an evidence-based framework that identifies multi-level intervention factors that influence implementation effectiveness, to interpret stakeholder feedback [37]. Over an 8-month period (September 2019 – May 2020), we engaged a broad range of stakeholders in the adaptation of the *First 1,000 Days* program to be father-inclusive. Our engagement efforts informed the design of a prospective randomized controlled trial enrolling the mother-father-infant triad beginning in pregnancy and continuing throughout the first year of life (Fig. 1).

Our engagement plan consisted of two components: community stakeholder engagement and qualitative interviews with new fathers. We chose each component to provide unique perspectives relating to issues such as father receptivity to program participation, study design, and intervention structure and content. Based on our prior work with *First 1,000 Days*, we also recognized that embedding our intervention within the larger community and gaining institutional support is critical for increasing the likelihood of intervention success [32].

### Setting

We directed our engagement efforts to include fathers and clinical leaders who receive and provide care at obstetric and pediatric practices affiliated with Massachusetts General Hospital (MGH) in Boston, Massachusetts. MGH includes hospital- and community-based care locations and serves a diverse population, with over 40% of delivering mothers identifying as a racial or ethnic minority. We aimed for our engagement sample to reflect this diversity. We also engaged community leaders with experience in engaging new parents, especially fathers, in early life interventions and connecting families with community resources.

### Community stakeholder engagement

We conducted a two-stage process of community engagement. We held the open Community Engagement Meeting (CEM) to introduce the study to community stakeholders. Following the meeting, we invited interested attendees to participate in a Community Advisory Board (CAB) to provide ongoing input on study design and father engagement.

#### Community Engagement Meeting (CEM)

We held an open CEM in September 2019 to guide the initial formative stages of intervention adaptation. In identifying meeting invitees, we leveraged existing community connections from the *First 1,000 Days*, as well as solicited requests from these connections to identify any other key stakeholders we may have overlooked. We systematically created an invite list, including MGH obstetric and pediatric clinicians providing care in the perinatal period, care providers from community home visiting programs, leaders of father advocacy groups, and local fathers. During this meeting, we provided an update on results from *First 1,000 Days*, explained our rationale in extending the program to include fathers, and described the current proposed intervention structure (Table 1). The initial intervention design was the product of an extensive literature review of effective obesity prevention and father-inclusive perinatal interventions targeting the first year of life [4, 38]. The research team collaboratively reviewed the current literature relation to existing *First 1,000 Days* intervention content to develop our proposed intervention structure.

**Table 1:** Specific Stakeholder Feedback in Relation to Initial “First Heroes” Intervention Protocol Design

	**Topic**	**Initial Protocol Design**	**Stakeholder Feedback**		
			***CEM***	***CAB***	***Interviews***
**Recruitment**	***Program branding***	“First 1000 Days Fatherhood Intervention”; obesity prevention initiative	Suggested “HEROES” name to capture the important/ inclusive role of fathers	N/A	N/A
	***Eligibility Criteria***	First time biological mother-father dyad, > 18 yo, English/Spanish speaking, intent to continue care at MGH obstetric and pediatric practice	Concern for excluding certain demographic groups (i.e. single/separated parents and same-sex couples)	N/A	N/A
**Intervention Design**	***Staffing***	Research nurse or Health Coach, supplemented by pediatrician and research assistant	-Staff team with sociodemographic diversity -Adequate educational backgrounds -Social skills/personality traits (non-judgmental, communicative, empathetic, flexible, trusting) -Not necessary to be healthcare professional but adequate training/supervision -Active role models (males and fathers)	-Train on cultural sensitivities and mandatory reporting -Pair (1) research nurse with academic experience and (2) health coach with community and parenting experience to provide well-rounded intervention delivery experience	Fathers open to a variety of different messengers to receive information relating to: -Their child’s health: trusted pediatrician (*n* = 6), their partner (*n* = 3), then family (*n* = 2) -Being a father: trusted other fathers (*n* = 4), pediatrician (*n* = 2), their own parents (*n* = 2), then family (*n* = 1) and peers (*n* = 1) -Their own health: trusted physician or another healthcare professional (*n* = 4), then peers (*n* = 2) and family (*n* = 2)
	***Content***	-Responsive parenting -Parent lifestyle behaviors -Access to resources/Social Determinants of Health	-Growth and developmental milestones -Specific supports for fathers to learn about infant temperament -Importance of social connectedness and relationships -Focus on post-partum mental health for both mothers and fathers	-Substance abuse information in relation to the emotional part of being a new parent -Frame as the importance of ‘being present’ for their child	-Basic routines (i.e. changing diapers) -Child sickness and medical emergencies -Critical developmental milestones -Early bonding with the baby -Supporting mothers through pregnancy and the postnatal period -Developing healthy and stress-reducing habits for parents
	***Visit timing/ associated critical time points***	Prenatal: 30–34 weeks gestation/3^rd^ trimester	Program initiation during pregnancy, when the decision to be an active parent occurs	N/A	Some fathers preferred program initiation before birth and some preferred after birth
		Postnatal 1: 3–4 weeks of age/establish feeding practices	N/A	N/A	-General support -Concern that 3–4 weeks was too late for urgent needs with breastfeeding support during weeks 1–2
		Postnatal 2: 3–4 months of age/introduction of solids	N/A	N/A	-General support (considered an “impactful time”) -Some thought too early for introduction of solids -Some suggested 3–4 months is too late because there is less uncertainty and fewer questions than during the first 1–2 months
	***Visit modality***	Prenatal: virtual visit Postnatal 1: home visit Postnatal 2: home visit	Consider some parents in target population cannot be in same place at the same time due to work or other conflicts	-Home visits over virtual visits, particularly when first meeting dyads to establish rapport; -Consider family preference	-Support for virtual prenatal visit -Preferred in-person home visits for postnatal visits
	***Delivery mode***	Print/web/text-based materials	N/A	-Package intervention content in ‘bite-sized’ pieces (videos, text messaging, and short summaries)	-Web-based materials -Mobile-based materials and printed information (especially postnatal period) -Advanced notice of topics and after-visit summaries
**Engagement**	***Engaging champions***	Job postings for Health Coach and Research Nurse positions distributed to CAB members, clinical champions with MGH (physician, nursing), local universities with graduate programs in public health, nursing, education, etc	N/A	-Use community health worker model for health coach position to address potential qualification barriers for applicants -Connect with professional associations and community organizations (i.e. Fathers’ Uplift, Nurturing Fathers Program, Roca) to further advertise positions	N/A
	***Engaging innovation participants***	Text messaging, videos, mobile applications, electronic groups	-Parent leaders from community partners to engage fathers -Dads in images for outreach -Focus group messages/images with men and women -Use incentives/ material goods	N/A	-Practical and evidence-based content -Low maintenance intervention -Home visitor continuity -Include peer and group support -Gift for participation -Adaptable/tailored to parental needs

#### Community Advisory Board (CAB)

We invited CEM attendees to provide ongoing feedback through participation in our CAB. We informed potential members that responsibilities would include (1) attending quarterly meetings and (2) providing feedback on intervention design and content. We asked members to identify other stakeholders within the fatherhood community for invitation. The first CAB meeting was held in January 2020. The meeting agenda addressed program modifications based on initial feedback, recruitment plans, and study educational materials. Board members received all study materials prior to the meeting for their review, with opportunities for feedback provided within the meeting as well as through follow-up phone conversations or written communication. The first meeting was held via video conference. To minimize the burden on our advisory board members during the COVID pandemic, we provided ongoing updates through email (Spring–Summer 2020), with resumption of the quarterly meeting schedule in Fall 2020.

### Qualitative interviews

#### Study design

We conducted 16 semi-structured qualitative interviews (November 2019-April 2020) with fathers of children under 1 year old to investigate the acceptability and feasibility of the proposed intervention. We identified fathers for participation through review of well-child visits with pediatric practices at MGH, including both hospital- and community-based locations. Fathers were eligible to participate if they were at least 18 years old, had a child receiving care at a MGH pediatric primary care site, were English proficient, were first time fathers, and had a child 0–12 months without significant medical comorbidities that would affect growth, development, and feeding. This study was approved by the MassGeneral Brigham Institutional Review Board.

#### Interview procedures

Study staff mailed recruitment letters to eligible fathers describing the engagement study. One week after the letters were mailed, study staff contacted fathers by phone to explain the study, answer questions, and enroll fathers who chose to participate. Three phone call attempts were made to reach each eligible father who received a letter. We called 137 fathers; 83 did not answer the phone, 17 declined, 21 were ineligible (*n* = 8 due to language barriers, *n* = 2 due to medical comorbidities, *n* = 3 due to child age > 12 months, *n* = 2 due to moving out of state, and *n* = 6 due to not being a first-time father), and 16 consented to participation. Participants received a $25 gift card upon interview completion. After providing informed consent, fathers participated in semi-structured, in-depth interviews. The development of the interview guide was informed by a review of prior studies exploring early life obesity prevention strategies [4] and literature review of relevant methodological considerations regarding father engagement [25, 27, 38, 39] as well as CFIR constructs [37]. The interview guide included core and probing questions to elicit discussion of relevant topics, such as fathers’ information and resource needs, perceptions of their roles and experiences, and preferences for intervention content and modalities (see Supplemental File). Each semi-structured 30-min phone interview was audiotaped and transcribed by an independent company for analysis. We reached thematic saturation with a total of 16 interviews, as review of transcripts revealed reinforcement of previously identified themes and no new themes were generated.

### Data analysis

We used the CFIR domains to organize feedback from community stakeholders as well as our thematic analysis of qualitative father interviews [37]. Two team members (RW, SS) organized stakeholder perceptions into relevant CFIR domains, including (1) characteristics of the intervention, relating to intervention advantages versus alternative solutions (relative advantage), potential implementation difficulties (complexity), and intervention design (design quality and packaging), (2) “outer setting” factors, relating to connections with other organizations (cosmopolitanism), (3) “inner setting” characteristics of the organization implementing the intervention, including norms and values (culture), and (4) characteristics of individuals involved in the intervention, including progress towards sustained intervention use (state of change), commitment to the program (identification), beliefs that they are capable of executing the intervention (self-efficacy), and other personal traits of both intervention participants and intervention staff (other attributes).

#### Community stakeholder meetings

At both the CEM and AB meeting, a research team member transcribed detailed notes of all feedback provided by meeting attendees. We reviewed findings in detail in group debrief meetings following both stakeholder meetings. We categorized transcribed notes into CFIR domains using a deductive approach.

#### Qualitative interview

We used an iterative immersion-crystallization inductive approach to conduct content analysis through repeated cycles of reading and discussing transcripts to identify predominant themes [40]. The full analysis team (HFM, RW, GK, MK, ET) individually read nine transcripts in-depth in sets of three before discussing as a group. Based on our initial list of themes, three team members (HFM, RW, GK) independently coded interview content line-by-line, collating codes into an Excel spreadsheet to generate a preliminary codebook. We reviewed independent coding for consensus between coders. We revised and reviewed the codebook after each set of three interviews.

After in-depth review of nine interviews with the full analysis team, we noted overall repetition of themes. We reviewed the codebook at this time, reorganizing all codes under relevant corresponding themes that had been identified through group discussion. Two coders (SS, RW) independently coded the next two interviews using the revised codebook, with agreement > 85%. The final five interviews were independently coded, with no new themes emerging from content review and discussion. We sorted codes within CFIR domains using a deductive approach.

## Results

### Stakeholder characteristics

For the Community Engagement Meeting (CEM), we invited 46 individuals to attend, representing MGH obstetric, pediatric, and research leadership (*n* = 18), obstetric and pediatric clinical champions (*n* = 4), local community and state programs focused on fatherhood or early childhood health (*n* = 17), community outreach/home visiting programs (*n* = 3), and fathers who were community leaders (*n* = 4). Ultimately, 22 invitees planned to attend and 11 attended; of those unable to attend, the primary reason was scheduling conflicts. Our CAB was primarily drawn from CEM attendees and was composed of 12 members, including representatives from pediatrics and obstetrics (*n* = 2), academic public health research (*n* = 1), community outreach/home visiting (*n* = 2), local family and community organizations (*n* = 4), state public health infrastructure (*n* = 1), and a national child health organization (*n* = 1) as well as a local father advocate (*n* = 1).

A total of 16 fathers completed the qualitative interview, with 8/16 receiving pediatric care at a community health center. Of participating fathers, 10/16 identified as white, 3/16 identified as Hispanic/Latino, and 3/16 identified as “other”. The majority of fathers had a college education or higher (10/16); the remainder had either completed high school/GED (*n* = 2) or some college (*n* = 4). The median age of participating fathers was 35 years (IQR: 32, 39).

### Stakeholder feedback

We present results through the five CFIR domains (*intervention characteristics, outer setting, inner setting, individual characteristics, and process*). Within each of these domains, we organize findings from community stakeholder meetings and qualitative interviews by mapping emerging themes to relevant CFIR constructs.

#### Intervention characteristics: key intervention attributes that influence implementation effectiveness

##### Relative advantage: perceived advantages of intervention relative to alternatives

At the CEM, attendees highlighted advantages that are unique to our intervention, including program initiation during pregnancy, specific outreach to fathers, and aim to empower both parents. Within the qualitative interviews, fathers identified several relative advantages of our proposed intervention, including convenient access to father-specific intervention content that was delivered directly to them as opposed to them seeking out on their own (Table 2).

**Table 2:** Father Engagement Interview Themes and Illustrative Quotes Mapped to CFIR Domains and Impact on Intervention Design: Intervention Characteristics

CFIR Construct	CFIR Construct Definition	Themes	Illustrative Quotes	Impact on Intervention Design
Relative Advantage	Stakeholders’ perception of the advantage of implementing the intervention versus an alternative solution	Perceived advantages of the proposed intervention: i. Unique father-specific content ii. Easy and convenient delivery iii. Individual (versus group) interaction and instruction	i. “I think having resources available for dads might make it more—might normalize more that dads are also involved in these decisions about parenting. That might increase dad involvement.” ii. “Getting more information and coming to the house is much easier than figuring out on your own where to go and who to talk you to.” iii. “To me, why I like it is it separates me from paying $500 to be amongst a group of 14 other people I don't know. That would shut me down from being as open and honest as I really want to be and need to be to learn. That’s a huge selling point in my opinion. That one-on-one is very beneficial.”	-Conscious integration of father-inclusive language to normalize involvement -Support for targeted outreach to fathers and one-on-one interactions
Complexity	Perceived difficulty of implementation, reflected by duration, scope, radicalness, disruptiveness, centrality, and intricacy and number of steps required to implement	Perceived barriers of the proposed intervention: i. Virtual visits: • Technological difficulties • In-person demonstration preferred ii. Home visits: • Intrusiveness • Tired/distracted parents iii. General intervention: • Scheduling conflicts • Disagreement with information presented	i. “People have different levels of comfort with technology and have different devices.” “I can tell you what wouldn’t be good virtually is lactation support… Somebody needs to show you. And I mean show your wife, and you be there.” ii. “I think for some families they probably feel uncomfortable with other people in their home or telling them what to do or what’s best for their baby.”; “at that stage of three or four weeks, we're not able to process, record, and make the information useful because we are tired or distracted.” iii. “Navigating any appointment ends up being navigating my wife's schedule, my schedule, the baby's, how the baby's doing. All of a sudden, anything becomes a little bit more complicated.”; “I may have different points of view of what they believe is correct.”	-Virtual visit tutorial available as needed; planned for in-person option but not available due to COVID19 -Reinforcement of visit materials through other materials (texts, emails, videos, printed materials) -Meeting families’ preferences relating to comfort with visits/content
Design Quality & Packaging	Perceived excellence in how the intervention is bundled, presented, and assembled	i. Preference for pre-and post-summary of visit topics ii. Mixed opinions regarding visit timing and structure iii. Diverse modalities of content delivery preferred iv. List of intervention topics are appropriate, with a highlighted need for information on healthy eating, sleeping, and activity for babies and parents	i. “I’m definitely the kind of person who want to receive the material ahead of time, I could actually read it, digest it, and ask questions instead of trying to absorb it during the visit.”; “If there’s a summary sheet of the key takeaways or key things to look out for, it’s always helpful.” ii. “One visit is probably not enough and two is—yeah I think that’s a perfect amount.”; “Maybe the first two visits are more closely scheduled, like we'll say 4 weeks, and then at 7 weeks, and then another one at 18 to 20 weeks.” iii. “I like videos, absolutely, and a web page. Maybe even a quick text with someone on the other line.”; “I would say printed information is good, but also maybe a link to the same type of information online would be helpful as well.” iv. “You’re hitting the nail on the head with every issue and period of the child’s growth that I think parents fear…Any way you can prepare parents with information before that will definitely put their mind at ease.”	-Preparation of “After Visit Summary” summarizing key content and dyad’s personal health goals – distributed after each Health Coaching Visit -Additional material (printed, texts) provided after 3–4 month health coach visit
Adaptability	The degree to which an innovation can be adapted, tailored, refined, or reinvented to meet local needs	Tailoring the intervention to specific parental needs encourages participation and engagement maintenance	“In each of those virtual or in-person visits, there has to be one real aha takeaway that I feel like, ‘Wow. I wouldn’t have thought of that,’ or, ‘That really added something to my parenting toolbox,’ rather than feeling like I was just getting some generic information.”	Ensuring that health coach visits are customizable to dyad priorities, don’t seem too “scripted”, with resources relevant to dyad social needs

##### Complexity: perceived difficulty of implementation

CEM attendees and CAB members reflected on ways in which the intervention must address the more complex sociocultural needs of a socioeconomically and racially diverse patient population such as through accommodating busy work schedules and training interventional personnel on cultural sensitivities and mandatory reporting (Table 1). Fathers identified several potential implementation barriers related to program delivery and content, such as scheduling conflicts, disagreement with content, technological difficulties, and intrusiveness of home visits (Table 2).

##### Design quality and packaging: how well the intervention is presented, bundled, and assembled

To brand the program in a way that immediately engages fathers, CEM attendees suggested an inclusive name for the program, with an emphasis on the theme of parents as ‘heroes.’ With regards to visit modality and delivery mode, both CAB members and fathers preferred home visits to virtual visits and recommended presenting intervention content in ‘bite-sized’ summaries before and after visits (Tables 1 and 2). CAB members suggested that key intervention messages be packaged in brief videos, text messages, or short summaries, while fathers expressed interest a “summary sheet of the key takeaways” with each visit. Both groups also recommended a degree of customization depending on dyads’ preferences.

Though we initially designed the visit structure and timing to align with critical developmental time points during the prenatal and postnatal periods, there were mixed attitudes amongst interviewees regarding the timing of each visit with respect to the pregnancy and child’s age as well as the overall structure of the proposed intervention (Table 1). CEM attendees, CAB members, and father interviewees generally supported the proposed intervention content. Stakeholders also proposed key content areas that they felt were important to include and highlight in the program curriculum, such as infant growth and development, as well as parental support for social connectedness, relationships, and mental health (Table 1).

#### Outer setting: factors external to the organization implementing the intervention

##### Cosmopolitanism: the overall connectedness with other organizations

To take advantage of existing resources that support new parents,, CEM attendees recommended connecting participants with local parenting, fatherhood, and child abuse prevention programs. Similarly, CAB members provided recommendations to relevant parenting and child development resources from national organizations, such as the National Institute for Children’s Health Quality [41], and local organizations, such as Boston Basics [42].

#### Inner setting: characteristics of the organization implementing the intervention

##### Culture: the organization’s norms, values, and assumptions

CEM attendees urged us to promote an internal culture that expects dads to be involved, thereby motivating fathers to participate in the intervention. Intervention activities should reinforce the value that dads are important in their children’s lives. CEM attendees also suggested including ways to show new fathers that they are not alone, such as through testimonials from other fathers and/or connecting fathers in support groups.

#### Characteristics of Individuals: qualities of individuals involved in the program

#### Participants

##### Individual State of Change: individuals’ progress towards enthusiastic and sustained use of the intervention

CEM attendees cautioned that many of our potential participants may not yet fully understand what it means to be a parent and may have lacked parenting role models within their own lives. As such, a goal of our project is to empower new parents in understanding their roles, moving them into a higher “state of readiness” to prepare to meet the needs of being a parent.

##### Identification with the organization: individuals’ relationship and commitment to an organization

CEM attendees highlighted the importance of building genuine relationships between the coaching team and parents. Strong relationships between the health coach and fathers will cultivate trust and keep the father engaged throughout the intervention.

##### Knowledge/beliefs about the intervention: individuals’ value placed on intervention

Father interviewees recognized a need for the proposed intervention and expressed they would like to be included with mothers when receiving information about parenting and infants (Table 3). Despite the diverse sources that dads-to-be draw on for support and advice, including family, clinicians, friends, and published information, the information they receive is often unclear, contradictory, and explicitly directed at mothers. Consequently, fathers discussed feeling largely unprepared with the information and skills necessary to support their babies and partners. Sleep disturbance and constant work were cited as the most physically draining aspects of being a new father. Emotionally draining challenges included the uncertainty and novelty of fatherhood, feeling of helplessness, relationship strain with the mother, and baby colic.

**Table 3:** Father Engagement Interview Themes and Illustrative Quotes Mapped to CFIR Domains and Impact on Intervention Design: Characteristics of Individuals

CFIR Construct	CFIR Construct Definition	Themes	Illustrative Quotes	Impact on Intervention Design
Knowledge & Beliefs about the Intervention	Individuals’ attitudes toward and value placed on the intervention as well as familiarity with facts, truths, and principles related to the intervention	Fathers perceive the intervention as needed and valued, based on: i. Their unique challenges preparing for and navigating fatherhood ii. Lack of targeted resources to support them with challenges and engage in parenting	i.“Where the mother is carrying the baby for nine months, she's gonna get it, you know, it's just, like, a natural reaction. Whereas the father's, like, oh my God, I have a new child. What do I do?”; “Anything I receive on becoming a dad, any advice. It just continues to get more difficult.” ii. “So many things are geared towards moms. Even when you read books, they are written for moms. It’s very clear. And that kind of makes you feel, as a father, like excluded.”; “I think having resources available for dads might make it more—might normalize more that dads are also involved in these decisions about parenting. That might increase dad involvement.”	-Additional support for conscious engagement of fathers through program materials -Specific content on importance of fathers
Self-efficacy	Individual belief in their own capabilities to execute courses of action to achieve implementation goals	Fatherhood is a rewarding and empowering experience, instilling a sense of purpose and self-efficacy to provide for the child and partner	- “It's been a rewarding new experience watching a little family member grow and mature and flourish.” - “I think the easiest thing is just being able to love my daughter. It's my first baby, and everything is all new to me, but it's—being a dad, I think just being able to love and hold and comfort my daughter is the easiest thing.” - “What do I like most about being a dad? The feeling of knowing that I have someone to come home to and what I’m to doing to provide for them.” - “The most important thing is just obviously I want her to be happy so whatever I can do to support her in that sense, I’m gonna do it.”	-Inclusion of developmental milestones and father-infant bonding within program materials -Included quotes from interviewed fathers within intervention to normalize father involvement
Other Personal Attributes	A broad construct to include other personal traits such as tolerance of ambiguity, intellectual ability, motivation, values, competence, capacity, and learning style	Additional personal attributes relevant to the intervention: i. Effects of fatherhood on father’s own health ii. Fathers’ perceived Parenting roles (day-to-day child-care, emotional support, and practical support)	i. “I have less time for gym or exercise. Not as much time as I used to have.”; “Taking care of yourself during that period is often hard because you’re just—that’s a secondary consideration, so I didn’t sleep much. I was eating at weird times. I was eating weird foods. Taking care of yourself was harder than taking care of your kid, actually.” ii. “Parenting is a two-person job. Just’cause your wife’s breastfeeding doesn’t mean you’re not involved in the nutrition of your child. Yeah, the earlier you’re involved, the happier you’ll be.”; “Just making sure that she’s healthy, so making sure that she’s developing, getting bigger and eating and monitoring to make sure she doesn’t have a fever.”; “I do a lot of the day-to-day on the home, everything around the home, cooking, cleaning…Mine’s more home and home maintenance, day-to-day maintenance.”; “A lot of it was just being proactive on all sorts of responsibilities more broadly than baby care, doing shifts as night so Mom can get some sleep.”	-Supported focus on paternal health behaviors, specifically related to nutrition, sleep, physical activity -Reinforced fathers’ role in infant caretaking

##### Self-efficacy: individuals’ belief in capacity to achieve implementation goals

CEM attendees spoke to the importance of messaging that fathers can make a difference in their children’s health. Supporting this theme, attendees framed fathers as “heroes”, suggesting that “all men want to be heroes to their child…if you include them, they will rise to this level.” Fathers bolstered the notion of self-efficacy, emphasizing the intrinsic motivation to provide for their child and partner (Table 3).

##### Personal attributes: traits of participating individuals

Within the CEM, attendees raised concern about the intervention inadvertently excluding certain demographic groups (Table 1). Fathers discussed ways in which the physical and mental strain of fatherhood adversely affect their personal health and the difficulties they faced in maintaining healthy self-care habits during the postnatal period. Highly relevant to the intervention and addressing these challenges is fathers’ perceptions regarding their main parenting roles and the importance of these roles (Table 3).

#### Intervention Personnel

##### Personal attributes

CEM attendees and CAB members provided suggestions on optimal skills and credentials we should seek in intervention staff, including sociodemographic diversity and a balance of social skills and personality traits with appropriate educational background, training, and supervision (Table 1). However, CAB members cautioned against too stringent educational requirements, as this may be a barrier for finding well-suited candidates from the community. Fathers were generally open to a variety of intervention staff delivering intervention content related to their child’s health, being a father, and their own health (Table 1).

#### Process: critical stages of program implementation

##### Engaging: involving appropriate individuals in the implementation and use of the program

*Champions (individuals who support program implementation):* To support recruitment efforts in hiring a health coach, CAB members recommended leveraging both local professional and community organizations to advertise the position. Given concern about educational requirements highlighted above, they suggested that using a community health worker (CHW) model may overcome this, as the CHW model recognizes the value of non-clinical skills, including lived experiences and connection with the target community (Table 1) [43].

*Innovation participants (individuals who participate in the program):* CEM attendees discussed the importance of engaging fathers directly through addressing their backgrounds, “meeting them where they are”. They suggested several outreach strategies to achieve this (Table 1). Fathers recommended a range of additional facilitators to recruitment and engagement maintenance (Table 1). Fathers also underscored the importance of adaptability (*degree to which that an intervention can be modified to individual needs*) (Table 2).

## Discussion

In the pre-implementation phase of the *First Heroes* randomized controlled trial, we used a structured process of multidimensional stakeholder engagement to adapt a mother-focused perinatal obesity prevention intervention to include fathers as equal participants. This process was instrumental in reinforcing community ties and increasing our understanding of fathers’ needs, strengthening our intervention to deeply engage fathers throughout the entire process. CFIR provided a framework for understanding and applying our stakeholders’ feedback. Our process demonstrated the value of including multiple perspectives when engaging stakeholders, as community leaders and new fathers provided insights that were both unique as well as mutually reinforcing.

While we were open to significant changes in our overall design based on feedback, our stakeholders instead highlighted key areas of focus that strengthened our planned intervention. Both community stakeholders and new fathers had strong support for our approach, citing the advantage of and need for parenting programs that include fathers and begin during pregnancy. Stakeholder input influenced our intervention values, delivery strategies, personnel, and content; we outline specific contributions in each of these domains below. Notably, there were no components of our proposed intervention that were eliminated or de-emphasized based on stakeholder feedback.

Our community stakeholders encouraged an inclusive culture that engages fathers from the start. We named our program *First Heroes*, uniting the preceding *First 1,000 Days* intervention with themes that arose in the CEM. Community stakeholders strongly believed fathers would rise to the expectations set for them. This was reinforced by the fathers we interviewed who spoke freely and candidly about the rewards and challenges of fatherhood, as well as interest in our program, if it was responsive to their needs.

Stakeholder engagement also influenced our program delivery strategies. We took feedback into account as we decided to allow participant preference to determine both visit type as well as options for receiving materials, as there was a clear interest among fathers for an intervention that could be tailored to their needs. We created materials that could be disseminated through a variety of modalities (e.g. print, email, text messaging). Materials were designed to be easily consumed and not burdensome (i.e. “bite-sized” content), including brief overviews of printed content and short videos summarizing key messages. Of note, based on feedback, we had decided to allow the choice of virtual versus home health coaching visits. However, due to the COVID19 pandemic, home visits were no longer an option and all health coaching visits have been conducted virtually.

Our community stakeholders emphasized essential qualities for the individual delivering our intervention, namely compassion and ‘soft’ skills that might not be able to be taught. Fathers demonstrated overall flexibility in who they would trust for advice, reinforcing that individual qualities were more important than objective characteristics. We responded to this by creating a health coaching “team,” including a social worker, dietitian, and an experienced health coach, one of whom was male.

Working with stakeholders across multiple dimensions provided unique insights for our intervention content. Community stakeholders were more attuned with ‘outer setting’ resources to integrate into and support our intervention, as well as the need for awareness of the impact of social determinants of health on infant and parent wellbeing. Fathers were more concrete about their needs, especially related to parenting education, sleep, feeding, development, and sickness. Both agreed on the importance of the social and emotional needs of new parents, which we made a priority in our intervention content.

While the primary aim of our engagement work was to inform the development of an obesity prevention intervention that equally engages mothers and fathers, obesity prevention themes were seldom explicitly discussed among any of our stakeholders. Despite this, targets for obesity prevention were frequent topics of discussion. Fathers identified feeding their child and promoting healthy growth as a key role, which the literature supports as key roles for fathers [17]. Additionally, new fathers endorsed the challenges of maintaining their own healthy sleep, nutrition, and physical activity habits after becoming a father, all of which are potentially obesogenic behaviors. Lastly, community stakeholders emphasized the importance of social determinants of health as a foundational target for our intervention. This resonates with an equity approach to obesity prevention, which requires consideration of basic needs and societal inequities as an essential first step [44]. Our engagement efforts and success in eliciting these priorities represent a model for engaging fathers in the development of perinatal and obesity prevention efforts.

Lastly, our engagement interviews with fathers informed our recruitment strategies, as we recognized the importance of providing additional methods of outreach to mother-father dyads. While we still prioritizing active outreach, we added passive methods to increase study awareness, including printed flyers and posters. Eligible mothers also received messages via the Electronic Health Record that provided an opportunity to initiate the enrollment process online. Additionally, given the relative homogeneity of the interview sample in relation to race/ethnicity and education, we recognized the importance of purposive sampling in identifying eligible dyads to ensure a diverse study sample.

### Limitations

While we attempted to recruit a diverse sample through outreach within the community health centers, we unfortunately were not able to logistically conduct interviews in Spanish due to the costs of translating transcribed interviews. As a result, our sample was relatively homogenous with regard to race/ethnicity and educational background. However, even within this sample, fathers identified a great need for resources and father outreach. Feedback from our community stakeholders was critical in providing a voice for the fathers and families they work with, who we were unable to engage through more traditional research methods.

Given existing literature that highlights struggles with recruiting fathers to participate in research, we used active strategies, as opposed to passive methods, for recruitment. Despite our multifaceted strategy with both mailed and phone outreach, we were unable to reach the majority of eligible fathers. We hypothesize that this does not demonstrate a lack of interest but instead reflects the challenge of identifying effective routes to reach fathers.

Additionally, our engagement efforts highlighted the need for feedback from a more diverse group of fathers. We will continue to prioritize understanding our participants’ experiences as we implement our intervention. Implementation science methods, such as CFIR, provide resources for informing the translation of research findings into practice and we intend to continue this participant-engaged approach throughout our work.

Notably, our engagement process overlapped with the early stages of the COVID19 pandemic, which resulted in shifting priorities for new fathers as well as our advisory board members. We had limited success in recruiting fathers for interviews after the onset of the pandemic, and our advisory board members had new responsibilities in responding to the crisis. While we had planned to increase the presence of fathers within our advisory board, as well as the diversity of fathers within our interview sample, our target population included communities who were most impacted by the pandemic at that time. We will continue to prioritize outreach to this group through our continued work.

Given the pandemic, in-person health coach visits were no longer possible, and we moved all interactions (including recruitment) to virtual. Our initial advice from our advisory board to focus on social determinants of health became more salient following COVID19. The challenges associated with COVID19 reinforced our efforts to address social needs in our intervention through appropriate community-based referrals.

### Conclusion

Through our engagement process, we identified significant benefits for multidimensional stakeholder involvement. This engagement study gave voice to fathers throughout the design of an intervention in a perinatal health area that does not traditionally include them. Using a structured framework with CFIR allowed us to meaningfully improve our intervention, specifically relating to values, delivery, personnel, and content. Recognizing the value of stakeholder engagement, we will continue talking to and learning from fathers throughout subsequent phases of the *First Heroes* program in an iterative process that incorporates fathers in the fight against childhood obesity. Our work provides a practical model for other investigators in designing and adapting interventions to new populations, especially those overlooked through traditional research initiatives.

## Supplementary Information

**Additional file 1. **First 1,000 Days Fatherhood Program Interview Guide.

## Data Availability

The datasets used and/or analyzed during the current study are available from the corresponding author on reasonable request.

## Abbreviations

CFIR: Consolidated Framework for Implementation Research
CEM: Community Engagement Meeting
CAB: Community Advisory Board

## References

[1] Hales CM, Carroll MD, Fryar CD, Ogden CL (2017). Prevalence of Obesity Among Adults and Youth: United States, 2015–2016. NCHS Data Brief.

[2] Ogden CL, Carroll MD, Fakhouri TH, Hales CM, Fryar CD, Li X (2018). Prevalence of Obesity Among Youths by Household Income and Education Level of Head of Household - United States 2011–2014. MMWR Morb Mortal Wkly Rep.

[3] Woo Baidal JA, Locks LM, Cheng ER, Blake-Lamb TL, Perkins ME, Taveras EM (2016). Risk Factors for Childhood Obesity in the First 1,000 Days: A Systematic Review. Am J Prev Med.

[4] Blake-Lamb TL, Locks LM, Perkins ME, Woo Baidal JA, Cheng ER, Taveras EM (2016). Interventions for Childhood Obesity in the First 1,000 Days A Systematic Review. Am J Prev Med.

[5] Morgan PJ, Young MD, Lloyd AB, Wang ML, Eather N, Miller A, et al. Involvement of Fathers in Pediatric Obesity Treatment and Prevention Trials: A Systematic Review. Pediatrics. 2017;139(2). 10.1542/peds.2016-2635 PMC6200318 28130430

[6] Davison KK, Kitos N, Aftosmes-Tobio A, Ash T, Agaronov A, Sepulveda M (2018). The forgotten parent: Fathers' representation in family interventions to prevent childhood obesity. Prev Med.

[7] Surkan PJ, Dong L, Ji Y, Hong X, Ji H, Kimmel M (2019). Paternal involvement and support and risk of preterm birth: findings from the Boston birth cohort. J Psychosom Obstet Gynaecol.

[8] Redshaw M, Henderson J (2013). Fathers' engagement in pregnancy and childbirth: evidence from a national survey. BMC Pregnancy Childbirth.

[9] Kotelchuck M. The impact of father’s health on the reproductive and infant health and development. In: Grau Grau MB, HR; las Huera, M, editor. Engaged Fatherhood for Men, Families and Gender Equality: Healthcare, Social Policy, and Work Perspectives: Springer; 2021. p. 31–61.

[10] Shah PS, Knowledge Synthesis Group on Determinants of Preterm/Low Birthweight Births (2010). Paternal factors and low birthweight, preterm, and small for gestational age births: a systematic review. Am J Obstet Gynecol..

[11] Sarkadi A, Kristiansson R, Oberklaid F, Bremberg S (2008). Fathers' involvement and children's developmental outcomes: a systematic review of longitudinal studies. Acta Paediatr.

[12] Yogman M, Garfield CF and the Committee on Psychosocial Aspects of Child and Family Health. Fathers' Roles in the Care and Development of Their Children: The Role of Pediatricians. Pediatrics. 2016;138(1). 10.1542/peds.2016-1128 27296867

[13] Wong MS, Jones-Smith JC, Colantuoni E, Thorpe RJ, Bleich SN, Chan KS (2017). The Longitudinal Association Between Early Childhood Obesity and Fathers' Involvement in Caregiving and Decision-Making. Obesity (Silver Spring).

[14] Freeman E, Fletcher R, Collins CE, Morgan PJ, Burrows T, Callister R (2012). Preventing and treating childhood obesity: time to target fathers. Int J Obes (Lond).

[15] Fraser J, Skouteris H, McCabe M, Ricciardelli L, Milgrom J, Baur L (2011). Paternal Influences on Children's Weight Gain: A Systematic Review. Fathering: J Theory Res Pract Men Fathers..

[16] Wake M, Nicholson JM, Hardy P, Smith K (2007). Preschooler obesity and parenting styles of mothers and fathers: Australian national population study. Pediatrics.

[17] Khandpur N, Blaine RE, Fisher JO, Davison KK (2014). Fathers' child feeding practices: a review of the evidence. Appetite.

[18] Penilla C, Tschann JM, Deardorff J, Flores E, Pasch LA, Butte NF (2017). Fathers' feeding practices and children's weight status in Mexican American families. Appetite.

[19] Hall L, Collins CE, Morgan PJ, Burrows TL, Lubans DR, Callister R (2011). Children's intake of fruit and selected energy-dense nutrient-poor foods is associated with fathers' intake. J Am Diet Assoc.

[20] Davison KK, Franckle RL, Lo BK, Ash T, Yu X, Haneuse SJ (2021). Infant sugar sweetened beverage and 100% juice consumption: Racial/ethnic differences and links with fathers' consumption in a longitudinal cohort. Prev Med Rep.

[21] Davison KK, Haines J, Garcia EA, Douglas S, McBride B (2020). Fathers' food parenting: A scoping review of the literature from 1990 to 2019. Pediatr Obes.

[22] Neshteruk CD, Nezami BT, Nino-Tapias G, Davison KK, Ward DS (2017). The influence of fathers on children's physical activity: A review of the literature from 2009 to 2015. Prev Med.

[23] Morgan PJ, Young MD (2017). The Influence of Fathers on Children's Physical Activity and Dietary Behaviors: Insights, Recommendations and Future Directions. Curr Obes Rep.

[24] Young MD, Morgan PJ (2017). Paternal Physical Activity: An Important Target to Improve the Health of Fathers and their Children. Am J Lifestyle Med.

[25] Alio AP, Bond MJ, Padilla YC, Heidelbaugh JJ, Lu M, Parker WJ (2011). Addressing policy barriers to paternal involvement during pregnancy. Matern Child Health J.

[26] Lu MC, Jones L, Bond MJ, Wright K, Pumpuang M, Maidenberg M (2010). Where is the F in MCH? Father involvement in African American families. Ethn Dis.

[27] Davison KK, Gavarkovs A, McBride B, Kotelchuck M, Levy R, Taveras EM (2019). Engaging Fathers in Early Obesity Prevention During the First 1,000 Days: Policy, Systems, and Environmental Change Strategies. Obesity (Silver Spring).

[28] Levy RA, Kotelchuck M. Fatherhood and Reproductive Health in the Antenatal Period: From Men’s Voices to Clinical Practice. In Grau Grau M, Bowles HR, las Huera M (eds). Engaged Fatherhood for Men, Families and Gender Equality: Healthcare, Social Policy, and Work Perspectives: Springer; 2021. p. 111–37.

[29] Bowles HR, Kotelchuck M, Grau Grau M. Reducing Barriers to Engaged Fatherhood: Three Principles for Promoting Gender Equity in Parenting. In Grau Grau M, Bowles HR, las Huera M (eds). Engaged Fatherhood for Men, Families and Gender Equality: Healthcare, Social Policy, and Work Perspectives: Springer; 2021. p. 299–325.

[30] Fleurence R, Selby JV, Odom-Walker K, Hunt G, Meltzer D, Slutsky JR (2013). How the Patient-Centered Outcomes Research Institute is engaging patients and others in shaping its research agenda. Health Aff (Millwood).

[31] Frank L, Forsythe L, Ellis L, Schrandt S, Sheridan S, Gerson J (2015). Conceptual and practical foundations of patient engagement in research at the patient-centered outcomes research institute. Qual Life Res.

[32] Blake-Lamb T, Boudreau AA, Matathia S, Tiburcio E, Perkins ME, Roche B (2018). Strengthening integration of clinical and public health systems to prevent maternal-child obesity in the First 1,000 Days: A Collective Impact approach. Contemp Clin Trials.

[33] Blake-Lamb T, Boudreau AA, Matathia S, Perkins ME, Roche B, Cheng ER (2020). Association of the First 1,000 Days Systems-Change Intervention on Maternal Gestational Weight Gain. Obstet Gynecol.

[34] Simione M, Moreno-Galarraga L, Perkins M, Price SN, Luo M, Kotelchuck M (2021). Effects of the First 1000 Days Program, a systems-change intervention, on obesity risk factors during pregnancy. BMC Pregnancy Childbirth.

[35] Taveras EM, Perkins ME, Boudreau AA, Blake-Lamb T, Matathia S, Kotelchuck M, et al. Twelve-Month Outcomes of the First 1000 Days Program on Infant Weight Status. Pediatrics. 2021;148(2). 10.1542/peds.2020-046706 PMC8579422 34326179

[36] Woolf SH, Zimmerman E, Haley A, Krist AH (2016). Authentic Engagement Of Patients And Communities Can Transform Research, Practice. And Policy Health Aff (Millwood).

[37] Damschroder LJ, Aron DC, Keith RE, Kirsh SR, Alexander JA, Lowery JC (2009). Fostering implementation of health services research findings into practice: a consolidated framework for advancing implementation science. Implement Sci.

[38] Lee JY, Knauer HA, Lee SJ, MacEachern MP, Garfield CF. Father-Inclusive Perinatal Parent Education Programs: A Systematic Review. Pediatrics. 2018;142(1). 10.1542/peds.2018-0437 29903835

[39] Vollmer RL, Adamsons K, Mobley AR (2019). Recruitment, Engagement, and Retention of Fathers in Nutrition Education and Obesity Research. J Nutr Educ Behav.

[40] Borkan J, Crabtree B (1999). Immersion/Crystallization. Doing Qualitative Research.

[41] Safe Sleep and Breastfeeding [Available from: www.nichq.org]. Accessed 25 April 2022.

[42] Boston Basics [Available from: https://boston.thebasics.org].. Accessed 25 Apr 2022.

[43] Brownstein JN, Hirsch GR, Rosenthal EL, Rush CH (2011). Community health workers "101" for primary care providers and other stakeholders in health care systems. J Ambul Care Manage.

[44] Kumanyika SK (2019). A Framework for Increasing Equity Impact in Obesity Prevention. Am J Public Health.

