# Supplementary material for: Stakeholder engagement in developing a father-inclusive early life obesity prevention intervention: First Heroes
Source: BMC Pregnancy Childbirth. 2022 May 27;22:443. doi: 10.1186/s12884-022-04759-z (PMC9145160; doi:10.1186/s12884-022-04759-z)
Supplement: Supplementary file 1 — Additional file 1. First 1,000 Days Fatherhood Program Interview Guide. [file 12884_2022_4759_MOESM1_ESM.docx]

**First 1,000 Days Fatherhood Program Interview Guide**

Thank you for agreeing to participate in an interview for the new childhood obesity prevention program. This program will support fathers and their families during the first 1,000 days period, which starts in pregnancy and continues until a child turns 2 years old. It will launch at MGH in early 2020, and the goal is to provide fathers with the support they need to help their children grow healthily from the start and create healthy lifestyles for their entire families. This program is being funded by the Maternal and Child Health Bureau.

Your participation in this interview is voluntary. The interview will last approximately 30 minutes, and you will receive a $25 gift card as a thank you for your time. You may choose not to answer any question for any reason. You may also end the interview at any time without consequence. With your permission, I will audio record our conversation. The conversation will then be transcribed for evaluation by a professional transcription service. Please do not use your name or any other identifying information during the recording. If we use the information you share in a publication or for a future training program, we will do so in a way that you cannot be personally identified, and your name will not be used.

Do you have any questions before we begin?

Thank you for participating in the interview. I am now documenting your consent to proceed.

Documentation of Verbal Consent

Date: __________ Time: ___ ___ : ___ ___ AM / PM

Interviewer Initials: ___ ___

(For remuneration/identifying only-to be saved on locked file)

Random ID_____________________________________

Name_________________________________________

Address ________________________________________

[TURN ON RECORDER]

The random ID for this recording is [RANDOM ID]

**Introduction**

Thank you so much for agreeing to do this interview. We really appreciate your time. We are asking you to do this interview because you are a father with a child under the age of one. Since you just went through this, we are interested in your thoughts. We are planning a new program that supports new dads during this time, and your feedback will help us make sure that our program is best directed to dads’ needs.

**Introductory Questions**

To begin, I’d like to talk a bit about you and your child.

1. What has been the easiest thing about being a dad?
2. What has been hardest thing?
3. What have you liked most about being a dad?
4. Thinking back, is there any advice you wish you had received before becoming a dad?
5. If you have questions about being a dad or taking care ofyour child, what do you do?

Probe: Do you go anywhere to get answers to these questions? *For example: friends, family, TV, Internet, books, magazines, newspapers, Facebook, mobile apps, and other types*

- 1. What kind of questions have you had?

**General Intervention Questions**

Now, we are going to move on to talking about the program that we are creating. The program will support dads and moms beginning before their baby is born and will continue through the first year of the baby’s life. The program will focus on things we know are important for everyone in the family to live a healthy life such as eating well, being physically active, getting enough sleep, and accessing support resources.

1. Let’s pretend that your child hasn’t been born yet and you have signed up for this program.
   1. What information would be **most important to you** that the program includes?
   2. How would you have wanted to get this information **before** the baby is born?

*Probe: printed materials, educational videos, social media, texts, emails, online materials, mobile apps, home visits, clinic visit, video calls, telephone calls, health professional visit*

- 1. How would you have wanted to get this information **after** [your child’s name] was born? *Probe: printed materials, educational videos, social media, texts, emails, online materials, mobile apps, home visits, clinic visit, video calls, telephone calls, health professional visit*
  2. Do you think that you would want to get information in a different way than your child’s mother? How so? *Probe: For example, are you more/less interested in receiving texts, using apps, having printed materials than your child’s mother?*

**Focus of Mother- vs. Father-Targeted Materials**

We know that both dads and moms are important to how kids grow and develop, but sometimes parenting resources may seem like they are designed with only moms in mind.

1. If you and your child’s mother are both getting information related to healthy habits for your child at the same time, how might you **want** the information you are receiving to be different from what your child’s mother receives?
2. If there were information designed specifically for dads about developing healthy habits with their kids, what do you think that information should focus on?
   1. How do you think that information might be different than what moms might get?
3. As your child is growing and developing, what do you think is different about their relationship with you compared to their mother?
   1. Does this change what information you might want to receive?
4. Before your child was born, what things were important to you in preparing to be a dad?

*Probe if needed – ways to support mom, ways to prepare for baby*

1. Now that your child is born, what do you see as the most important things that you do in taking care of your child?
2. What do you do to help your child live a healthy lifestyle?
3. What do you do for yourself to stay healthy?
4. When was your last check-up, also known as a physical, with your own doctor? When is your next one?
5. Did you think about having a check-up with your doctor before your baby was born? Why?
   1. If no: Looking back, would you have wanted to? Why?

*Probe: What would have motivated you? What would have made it easier?*

**Specific Intervention Components**

Thank you so much for the feedback you have shared with me so far. Now, we are going to talk about what the program we are creating might look like. We have a few ideas, and we are interested in what you think. It’s okay to be honest and tell us if something does not sound like a good idea, or if it would not work for your family.

*Prenatal Intervention*

1. What if we offered new moms and dads a virtual visit to help prepare them for being parents before their baby is born. A “virtual” visit would mean a video call with an expert on healthy habits for parents and babies.
   1. What would you think about this?
2. In this visit, we would plan to talk about healthy habits for moms and dads relating to sleep, nutrition and physical activity. We would also talk about preparing for feeding and soothing babies, and babies’ sleep patterns after they are born.
   1. What do you think about these topics?
   2. Is there anything else you think would be important to address?
   3. What about a virtual visit would you find helpful?
   4. What would make it challenging to do a visit like this?
   5. If you had a visit like this…
      1. Are there any materials you would like to receive before the visit?

*Probe: printed materials, access to website, mobile app, emails, texts*

- - 1. Are there any materials you would like to receive after the visit?

*Probe: printed materials, access to website, mobile app, emails, texts*

- 1. Is there anything specific for fathers you would want this visit to include?

*First Home Visit*

1. We are also thinking of doing a visit home visit with both mom and dad when the baby is between 3-4 weeks old? At this visit, an expert on healthy habits would go over healthy growth feeding, sleep, and playtime for babies, as well as healthy habits for dad and mom. They would also talk about adjusting to being a parent, and where to get support if new parents need it.
   1. What do you think about this?
   2. Would you find it useful?
   3. What would make it more helpful?
   4. Are there any other topics you would want to include?
   5. Is there anything specific for fathers you would want this visit to include?
   6. Do you have any concerns about having a home visit?
   7. What might make it difficult to visit at home when they baby is 3-4 weeks old?
   8. If you had a visit like this…
      1. Are there any materials you would like to receive before the visit?

*Probe: printed materials, access to website, mobile app, emails, texts*

- - 1. Are there any materials you would like to receive after the visit?

*Probe: printed materials, access to website, mobile app, emails, texts*

*Second home visit.*

1. We also plan to do a second visit with both mom and dad when the baby is between 3-4 months old. The visit would on healthy growth, starting to give babies solid food, healthy sleep patterns, and ideas for playing with babies. We would also check in on how mom and dad are doing and connect parents to resources if they need them.
   1. This visit would either be done as a virtual visit using a video call (like the visit before the baby is born) OR a home visit (like the visit after the baby is born). Between these two options:
      1. Is there an option you prefer?
      2. Why?
   2. Would you find a visit at 3-4 months useful?
   3. What would make it more helpful?
   4. What might make it difficult to do a visit like this at this time?
   5. Are there any other topics you would want to include at this visit?
      1. Are there any materials you would like to receive before the visit?

*Probe: printed materials, access to website, mobile app, emails, texts*

- - 1. Are there any materials you would like to receive after the visit?

*Probe: printed materials, access to website, mobile app, emails, texts*

- 1. Is there anything specific for fathers you would want this visit to include?

*Other Engagement*

Thank you so much for your open and honest feedback so far. All of the information you have provided so far will help us make sure that the program we develop is designed to best meet dads’ needs.

In the ideas we talked about above, there would be three visits with both mom and dad – the virtual visit before the baby is born and two visits after the baby is born.

1. What do you think about this visit structure?

*Probe: Does it sounds like too little? Too much?*

We think it is important to provide information specifically for dads outside of those visits too.

1. Do you think that would be helpful? Why or why not?
2. What would be the best way to have access to more information and support in between these visits?

*Probe: printed materials, access to website, mobile app, emails, texts, phone calls*

Now think about the whole program- a virtual visit in pregnancy, two visits after the baby is born, and additional information provided in between visits. If you had the choice to sign up for a program like this. What would be important in **helping you decide whether or not to sign up**?

- 1. What would make you more likely to sign up?
  2. What would make you less likely to sign up?
  3. What might be hard about being part of a program like this?

1. Now let’s pretend you were part of a program like this. What would be important in **keeping you interested in the program**?
2. What if we were to provide small gifts, such as a toy or a piece of clothing, for your baby. What types of small gifts would you want to receive and when?

*Who is the messenger of advice?*

Thank you so much for all of your time so far. We are almost done but have a few more questions to cover. We are interested in learning who you trust most in getting information now that you are a dad.

1. If you are getting information **about how to help your baby start with healthy habits related to nutrition, sleep, and activity from a young age**, who would you trust?
   1. How would it matter to you if the person is a man or a woman?
   2. How would it matter to you if the person is a father or not a father?
   3. How would it matter to you if the person has the same cultural background?
   4. How would it matter to you if the individual is a health care professional?
   5. *Probe if needed for the above – why or why not?*
2. If you are getting information about **how to be a father**, who would you trust?
   1. How would it matter to you if the person is a man or a woman?
   2. How would it matter to you if the person is a father or not a father?
   3. How would it matter to you if the person has the same cultural background?
   4. How would it matter to you if the individual is a health care professional?
   5. *Probe if needed for the above – why or why not?*
3. If you are getting information **about how to take care of your own health**, who would you trust?
   1. How would it matter to you if the person is a man or a woman?
   2. How would it matter to you if the person is a father or not a father?
   3. How would it matter to you if the person has the same cultural background?
   4. How would it matter to you if the individual is a health care professional?
   5. *Probe if needed for the above – why or why not?*

**Demographic Characteristics**

Lastly, We have a few final questions to help us better understand you and your family.

1. What is your age?
2. Are you currently living with your child?
3. What is your race/ethnicity?
   1. Do you consider yourself Hispanic/Latino?
4. What is your highest level of education?
5. Dp you know if your family is eligible for WIC?
6. If you have any, what kind of health insurance do you have?

**We are thinking about forming a Dads Advisory Group as part of the study.** This would be a small group of dads we could contact a couple of times a year for ideas or feedback on what we are thinking about doing for families in our program.

Do we have permission to contact you if we have questions in the future?

**Closing**

This is the end of the interview. Thank you so much for your time. I really appreciate all that you have shared with me today. You will be receiving a $25 gift card in the mail as a thank you for your time.
